# Qiju Granule alleviates retinal damage in a mouse model of age-related macular degeneration

**DOI:** 10.3389/fmed.2026.1781217

**Published:** 2026-03-24

**Authors:** Qiang Chen, Jing Zhang, Kai Xu, Xinyu Liu, Lina Liang

**Affiliations:** Department of Eye Function Laboratory, Eye Hospital, China Academy of Chinese Medical Sciences, Beijing, China

**Keywords:** age-related macular degeneration, angiogenesis, apoptosis, Qiju Granule, retinal protection

## Abstract

**Background:**

Age-related macular degeneration (AMD) is the leading cause of visual loss in older people. Despite recent advances, the pathophysiology of AMD remains incompletely understood, and current therapies are limited. Qiju Granule is a traditional herbal formulation with therapeutic potential; however, its efficacy and mechanisms in AMD remain unclear.

**Objective:**

The aim of this study was to evaluate the protective effects of Qiju Granule in a mouse model of AMD and investigate potential underlying mechanisms.

**Methods:**

AMD-like retinal injury was induced in C57BL/6J mice by chronic light exposure combined with hydroquinone feeding. Retinal function and structure were assessed using electroretinography and histomorphological analyses. Retinal cell apoptosis and the expression of key angiogenic factors (VEGF and CD31) were assessed to explore underlying mechanisms.

**Results:**

Qiju Granule treatment significantly preserved retinal function, as reflected by improved electroretinographic responses, and maintained structural integrity of the retina and RPE layer. Mechanistically, Qiju Granule exerted potent anti-apoptotic effects, evidenced by a marked reduction in TUNEL-positive retinal cells. Furthermore, it downregulated VEGF and CD31 expression, indicating suppression of aberrant angiogenic signaling.

**Conclusion:**

Our findings identified a protective role of Qiju Granule in mitigating AMD-related retinal damage and suggested its potential as a therapeutic candidate to prevent progression to advanced AMD.

## 1 Introduction

Age-related macular degeneration (AMD) is a leading cause of irreversible central vision loss in individuals aged >55 years, with its global prevalence estimated at 8.69% of the population (196 million people) in 2020 and projected to reach 288 million by 2040 (1). As a progressive retinal degenerative disorder, AMD is clinically classified into dry (atrophic) and wet (neovascular) subtypes, with dry AMD accounting for approximately 90% of all cases (2). The core pathological features of AMD include retinal pigment epithelial (RPE) cell dysfunction, photoreceptor degeneration, drusen formation, and aberrant angiogenesis—driven by multifactorial processes such as oxidative stress, complement system activation, and choroidal capillary degeneration (3–6). While anti-vascular endothelial growth factor (anti-VEGF) therapies have transformed the management of wet AMD (7), there remains no approved disease-modifying treatment for dry AMD (8). Current interventions are limited to lifestyle modifications and nutritional supplements (e.g., AREDS formulations) (9, 10), which only slow disease progression without reversing retinal structural damage or preserving visual function, highlighting an urgent unmet clinical need for effective multi-target interventions (11).

Traditional Chinese medicine (TCM) has emerged as a promising strategy for chronic degenerative ocular diseases due to its holistic syndrome differentiation and multi-component synergistic effects (12–14). Qiju Granule is a TCM formulation derived from two classical prescriptions: *Shihu Yeguang Pill* (from the Yuan Dynasty’s Yuanji Qimi) and *Qiju Dihuang Pill* (from the Qing Dynasty’s Yiji: Miscellaneous Disease Formulas). Clinically, it is used for early-to-moderate dry AMD with the syndrome of liver-kidney yin deficiency, exerting therapeutic effects by nourishing the liver and kidney, replenishing yin, improving eyesight, and enhancing ocular microcirculation (15, 16). Previous preclinical work suggests that Qiju Granule alleviates retinal damage in a sodium iodate-induced rat model of dry AMD by upregulating neurotrophic factors such as basic fibroblast growth factor (bFGF) and brain-derived neurotrophic factor (BDNF) (17). Additionally, individual bioactive components of Qiju Granule—including chlorogenic acid from Chrysanthemum, puerarin from Puerariae Lobatae Radix, and proanthocyanidins from Sea Buckthorn—have been reported to possess antioxidant, anti-inflammatory, and retinal protective properties (18–21). These components align with the core pathological pathways of AMD, as they can inhibit oxidative stress-induced RPE cell damage, suppress hypoxia-mediated VEGF upregulation, and reduce retinal cell apoptosis—key processes implicated in AMD progression (3).

Despite these promising preliminary findings, existing preclinical research on Qiju Granule for AMD faces notable methodological and mechanistic limitations. A primary limitation is the reliance on acutely induced oxidative stress models, such as sodium iodate injection (17), which primarily model acute RPE necrosis rather than the insidious, progressive pathology characteristic of human AMD. These models fail to incorporate chronic environmental risk factors—including prolonged light exposure and exposure to smoking-related toxins such as hydroquinone (22)—that are central to AMD pathogenesis, and thus do not adequately replicate the gradual RPE dysfunction, photoreceptor degeneration, and incipient angiogenic activation that define early-to-intermediate stages of the human disease (7, 8, 23, 24). Furthermore, the mechanistic understanding of Qiju Granule’s efficacy remains incomplete, as prior investigations have predominantly focused on its antioxidant and neurotrophic properties while neglecting its potential impact on angiogenic signaling—a critical pathway in AMD progression. Key mediators such as VEGF and platelet-endothelial cell adhesion molecule-1, (PECAM-1/CD31; CD31) are pivotal drivers of the transition from dry AMD to the vision-threatening neovascular (wet) subtype (25–27); however, the regulatory effects of Qiju Granule on these angiogenic factors in the context of chronic retinal injury have not been systematically evaluated. Collectively, these gaps limit the translational relevance of existing data, as efficacy demonstrated in acute injury models may not predict therapeutic performance in the chronic, multifactorial setting of clinical AMD.

In this study, we established a murine model of early-to-intermediate AMD induced by chronic white light exposure combined with hydroquinone feeding—a model that closely mimics the core environmental risk factors and progressive retinal pathology (RPE damage, photoreceptor degeneration, and aberrant angiogenic signaling) observed in human AMD (2, 22). We systematically evaluated the protective effects of Qiju Granule on retinal structure and function using electroretinography and histopathological staining, and explored its underlying mechanisms related to retinal cell apoptosis and the regulation of VEGF/CD31-mediated angiogenic signaling. This research not only validates the therapeutic efficacy of Qiju Granule in a more clinically relevant AMD model but also expands the understanding of its multi-target regulatory mechanisms, providing critical experimental evidence to support its clinical application for early-to-intermediate dry AMD. Moreover, our findings address the unmet need for effective dry AMD interventions and lay a foundation for the development of TCM-based multi-target therapies for this debilitating ocular disease.

## 2 Materials and methods

### 2.1 Animals

Specific-pathogen-free (SPF) C57BL/6J mice (6–8 weeks old) were obtained from Beijing Vital River Laboratory Technology Co., Ltd. (Beijing, China). A total of 100 mice were assigned to five groups (*n* = 20/group): normal control group, AMD model group, Qiju Granule low-dose group, medium-dose group, and high-dose group. The mice were housed in a temperature-controlled environment with a 12-h light/dark cycle and given ad libitum access to food and water.

For all *in vivo* ocular examinations, mice were anesthetized by intraperitoneal injection of a ketamine/xylazine mixture (100 mg/kg ketamine HCl and 15 mg/kg xylazine HCl). Topical anesthesia was achieved with oxybuprocaine hydrochloride eye drops, and mydriasis was induced with 1% tropicamide as needed before examination. At the end of the observation periods (14 and 28 days), mice were deeply anesthetized (increased ketamine/xylazine dose) and euthanized by cervical dislocation, followed by immediate enucleation for histopathological and molecular analyses.

### 2.2 Reagents and instruments

Qiju Granule (catalog no. C-QJ-200901-P) was provided by Increasepharm Innovative Pharmaceutical Manufacturing Co., Ltd. (Zhuhai, Guangdong, China). Tropicamide Eye Drops (catalog no. MP2065) and Oxybuprocaine Hydrochloride Eye Drops (catalog no. B2092) were obtained from Santen Pharmaceutical Co., Ltd. (Osaka, Japan). Goat Anti-Rabbit Secondary Antibody (catalog no. ZF-0316) was sourced from Beijing Zhongshan Jinqiao Biotechnology Co., Ltd. (Beijing, China). Optimal Cutting Temperature Compound (OCT) (catalog no. 4583) was purchased from SAKURA Finetek (Torrance, CA, USA). Fluorescent Mounting Media (catalog no. C-101), Hematoxylin–Eosin (HE) Staining Kit (catalog no. C02-04004) were obtained from Beijing Biosynthesis Biotechnology Co., Ltd. (Beijing, China). 4′,6-Diamidino-2-phenylindole (DAPI) Staining Solution (catalog no. 564907) was purchased from BD Pharmingen™ (San Diego, CA, USA). Anti-VEGF Antibody (catalog no. ab68428) and Anti-CD31 Antibody (catalog no. ab108319) were provided by Abcam (Cambridge, Cambridgeshire, UK). Small animal visual electrophysiology instrument (D430) was obtained from Diagnosys LLC (Lowell, MA, USA). Small animal retina micro imaging system (OPTO-RIS-CN T3630) was provided by Optoprobe Science Ltd. (Beijing, China). Optical microscope (DM2500) and Cryostat (CM1850) were obtained from Leica Microsystems (Wetzlar, Hesse, Germany).

### 2.3 Drug preparation and administration

The Qiju Granule doses were determined based on the clinical adult dosage and standard human-to-mouse body surface area conversion. The clinical adult crude-drug dosage is 53 g/day (60-kg adult). Using a body surface area conversion coefficient (mouse:human = 0.081), the equivalent mouse dose was calculated as 9 g/kg/day (medium dose). The low (4.5 g/kg/day) and high (18 g/kg/day) doses were set to 0.5× and 2× the medium dose, respectively, to evaluate dose-dependent effects (28).

### 2.4 Model establishment and grouping

To establish the model, 80 mice were exposed to white light for 12 h/day (2,500 lux) and fed a diet supplemented with hydroquinone (0.8 g/kg) for 3.5 months. Subsequently, the mice were randomized into four groups (*n* = 20/group): model control and low-, medium-, and high-dose Qiju Granule. Twenty age-matched mice were maintained under standard housing and diet without intervention as the normal control group. Qiju Granule doses were 18, 9, and 4.5 g/kg (crude-drug equivalent) for the high-, medium-, and low-dose groups, respectively. Treatments were administered once daily by oral gavage for 14 or 28 consecutive days, depending on the time point. Model establishment was confirmed by reduced ERG amplitudes and histological evidence of retinal injury in the model control group compared with normal controls.

### 2.5 Electroretinogram (ERG)

Electroretinographic activity was assessed as previously described (29). After a 2-h period of dark adaptation, the mice were subjected to general anesthesia combined with local anesthesia and mydriasis. A specialized mouse corneal electrode was used, and the mice were placed on a warming device to maintain body temperature. Standard flash stimuli were used to elicit full-field ERG responses from the retinal. The frequency band used was 0.1–1 to 300–1,000 Hz, with a single stimulus applied. ERG measurements were performed five times, and the values were recorded for analysis.

### 2.6 Observation of RPE layer tissue structure

Mice were euthanized at the designated experimental time points by cervical dislocation, and their eyes were promptly enucleated and fixed in 4% paraformaldehyde. After 2 h, the anterior segment and a portion of the vitreous were carefully removed. The remaining ocular tissues were further fixed for an additional 24 h. Subsequently, the neural retina was gently peeled off, and retinal flat mounts were prepared. The structural integrity and damage degree of the RPE layer were observed and evaluated under a laser confocal microscope.

### 2.7 Histopathological observation

After deep anesthesia as previously described, mice were euthanized by cervical dislocation at the specified time points, with their eyeballs rapidly enucleated and immediately immersed in 4% paraformaldehyde solution for 2 h of fixation. Subsequently, the anterior segment and a portion of the vitreous were carefully dissected and removed. The remaining ocular tissues were further fixed in 4% paraformaldehyde for an additional 24 h, followed by dehydration through a graded ethanol series, embedding in paraffin wax, and continuous sectioning at a thickness of 4 μm. H&E staining was performed according to standard protocols to evaluate histopathological changes in the retinal tissue, with a particular focus on the structural integrity of photoreceptor cells and the RPE layer.

### 2.8 Terminal deoxynucleotidyl transferase dUTP nick end labeling (TUNEL) assay

Deeply anesthetized mice were euthanized by cervical dislocation. The isolated eyeballs were immediately immersed in 4% paraformaldehyde solution and fixed at 4 °C. After completion of fixation, the samples were embedded in OCT compound and snap-frozen in liquid nitrogen. Frozen retinal sections (8 μm in thickness) were prepared using a cryostat set at −23 °C. The TUNEL assay was performed to detect apoptotic cells according to the manufacturer’s instructions provided with the TUNEL kit. Following the TUNEL reaction, cell nuclei were counterstained with 4′,6-diamidino-2-phenylindole (DAPI). The sections were then mounted with anti-fluorescence quenching mounting medium and observed under a fluorescence microscope.

For quantitative analysis, eight slides were randomly selected from each group, and three random visual fields were chosen per slide to count the number of apoptotic cells and total cells. The apoptosis rate was calculated as the ratio of apoptotic cells to total cells in each field, and the average value was used for statistical analysis.

### 2.9 Immunofluorescence staining

Immunofluorescence staining was performed as previously described (17). Briefly, the 8 μm frozen retinal sections were first rinsed in phosphate-buffered saline (PBS) at room temperature for 10 min, and this washing step was repeated three times to remove residual OCT compound and fixative. Subsequently, the sections were incubated in 1% Triton X-100 solution for 30 min at room temperature to permeabilize the cell membranes, facilitating antibody penetration. After membrane permeabilization, nonspecific antibody binding was blocked by incubating the sections in 2% bovine serum albumin (BSA) solution for 2 h at room temperature. The sections were then incubated overnight at 4 °C with primary antibodies against VEGF and CD31. Following overnight incubation, the sections were washed three times with PBS at room temperature, 10 min per wash, to remove unbound primary antibodies. Next, the sections were incubated with rhodamine-labeled goat anti-rabbit secondary antibody for 1 h at room temperature in the dark to avoid fluorescence quenching.

After secondary antibody incubation, cell nuclei were counterstained with 4′,6-diamidino-2-phenylindole (DAPI) for 5 min at room temperature. Finally, the sections were mounted with anti-fluorescence quenching mounting medium to preserve fluorescence signals, and images were captured under a fluorescence microscope for qualitative and quantitative analysis of VEGF and CD31 expression.

### 2.10 Statistical methods

Statistical analyses were performed using SPSS 24.0 software for Windows. Measurement data were expressed as mean ± standard deviation (mean ± SD). For normally distributed data, one-way analysis of variance (ANOVA) was used to compare differences among multiple groups, followed by the least significant difference (LSD) *post-hoc* test for pairwise comparisons. For non-normally distributed data, non-parametric tests were applied: the Kruskal–Wallis H test was used for multiple group comparisons, and the Mann–Whitney U test was employed for pairwise comparisons. The chi-square test was utilized for analyzing categorical data to compare proportions between groups. A two-tailed *p* value < 0.05 was considered statistically significant.

## 3 Results

### 3.1 The effect of Qiju Granule on retinal function

**Figure 1:**
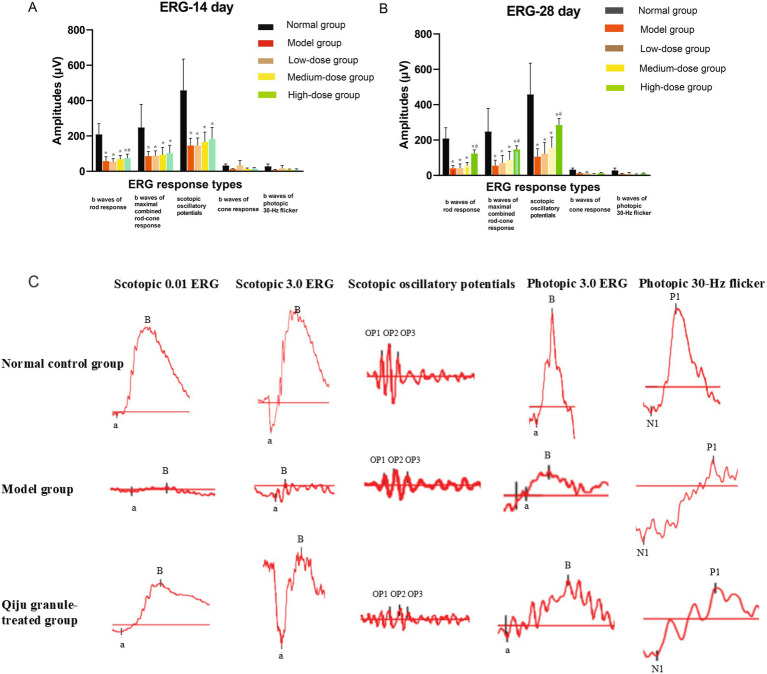
Full-field electroretinograms (ERGs) of mice subjected to different treatments. The figure presents quantitative changes in ERG amplitudes and representative waveform traces following treatment interventions. **(A)** Quantification of ERG amplitudes after 14 days of intervention. Data are shown for each experimental group (*n* = 10 per group). *p < 0.05 vs. the normal control group; #*p* < 0.05 vs. the model group. **(B)** Quantification of ERG amplitudes after 28 days of intervention. Data are shown for each experimental group (*n* = 10 per group). *p < 0.05 vs. the normal control group; #*p* < 0.05 vs. the model group. **(C)** Representative ERG waveform traces recorded from mice in each group at 14 and 28 days. ERG, electroretinogram.

As shown in Figure 1, notable alterations in ERG response amplitudes were observed following 14 days of intervention. Both the model group and Qiju Granule-treated groups exhibited reduced ERG amplitudes relative to the normal control group. Nevertheless, the high-dose Qiju Granule group displayed a marked elevation in rod photoreceptor response amplitudes compared with the model group.

After 28 days of treatment, all experimental groups presented a significant decrease in all detected ERG parameters when compared to the normal control group. Despite this overall downward trend, the high-dose Qiju Granule group still demonstrated significantly superior performance to the model group in terms of rod response, maximal combined rod-cone response, and scotopic oscillatory potential amplitudes (*p* < 0.05).

### 3.2 The effect of Qiju Granule on RPE layer structure

**Figure 2:**
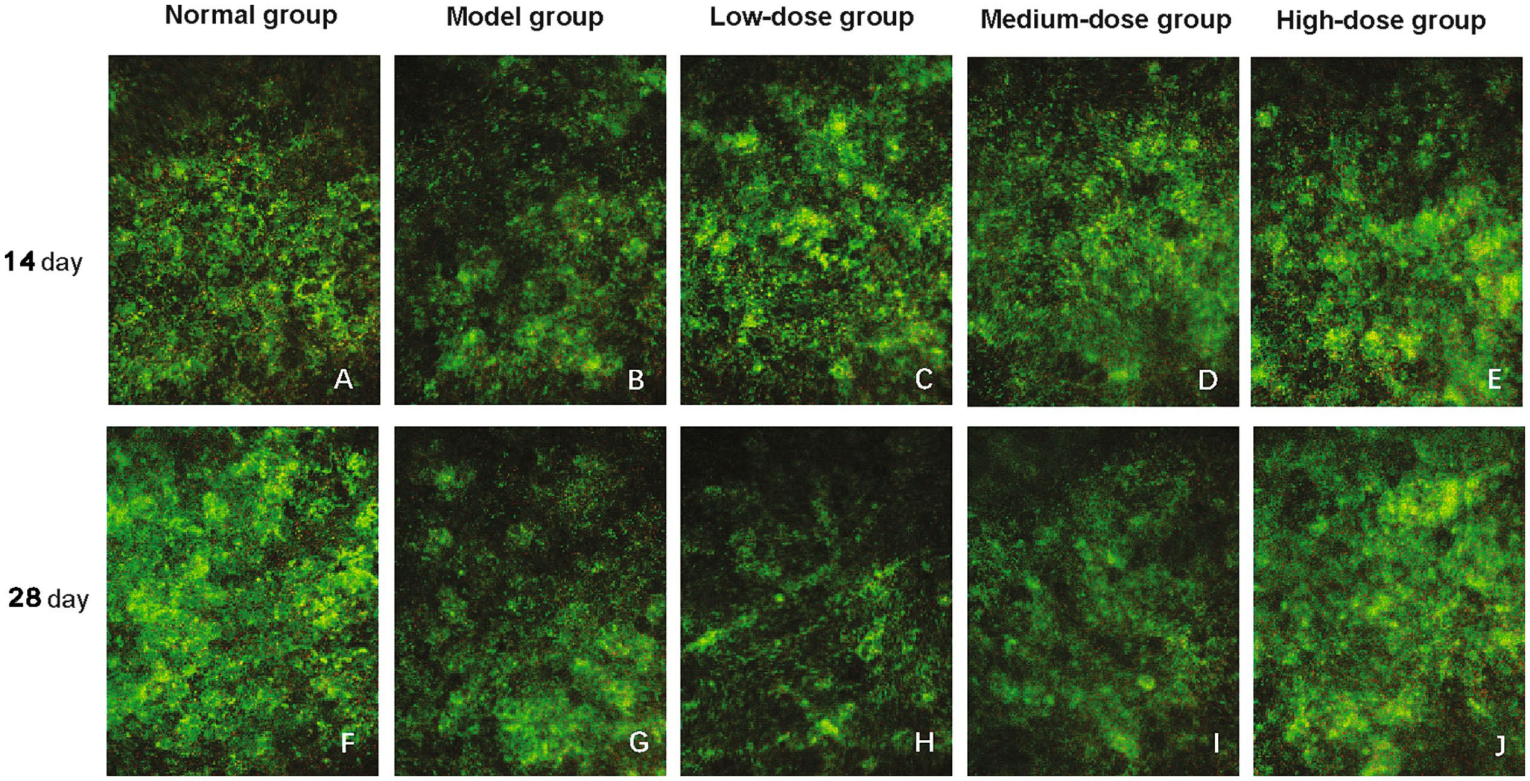
Histological observation of the retinal pigment epithelium (RPE) layer (magnification, 400×). The figure shows representative histological images of the RPE layer in mice from each group at 14 and 28 days, illustrating the morphological changes following treatment. **(A–E)** Images from the normal control group, model group, low-dose group, medium-dose group, and high-dose group, respectively, at 14 days post-intervention. **(F–J)** Images from the normal control group, model group, low-dose group, medium-dose group, and high-dose group, respectively, at 28 days post-intervention. RPE, retinal pigment epithelium.

The protective effect of Qiju Granule on the RPE layer was evaluated through observing the spontaneous fluorescence of RPE cells. In the normal control group, RPE cells exhibited orderly green spontaneous fluorescence arranged in a typical honeycomb pattern, with uniform granular fluorescent signals distributed in the cytoplasm (Figures 2A,F), indicating intact RPE layer structure.

After 14 days of intervention, the model group showed significantly attenuated spontaneous fluorescence intensity and disorganized arrangement of RPE cells, with the characteristic honeycomb structure disrupted (Figure 2B). The low-dose Qiju Granule group also presented reduced fluorescence intensity, with no obvious improvement in RPE cell arrangement (Figure 2C). In contrast, the medium-dose (Figure 2D) and high-dose (Figure 2E) Qiju Granule groups maintained spontaneous fluorescence intensity comparable to that of the normal control group, and partial regions retained the regular honeycomb structure of RPE cells, suggesting a protective effect on RPE layer integrity.

At 28 days post-treatment, the RPE layer damage in the model group further aggravated, showing a marked reduction in RPE cell number and scattered, uneven fluorescence distribution (Figure 2G). The low-dose Qiju Granule group displayed similar disorganized RPE cell patterns to the model group, with no significant structural improvement (Figure 2H). However, the medium-dose (Figure 2I) and high-dose (Figure 2J) Qiju Granule groups exhibited notably enhanced spontaneous fluorescence intensity, and the honeycomb intercellular connections were effectively preserved. These results indicate that Qiju Granule can protect the structural integrity of the RPE layer in a dose-dependent manner, with medium and high doses exerting more significant protective effects.

### 3.3 Qiju Granule ameliorates retinal histopathological changes

**Figure 3:**
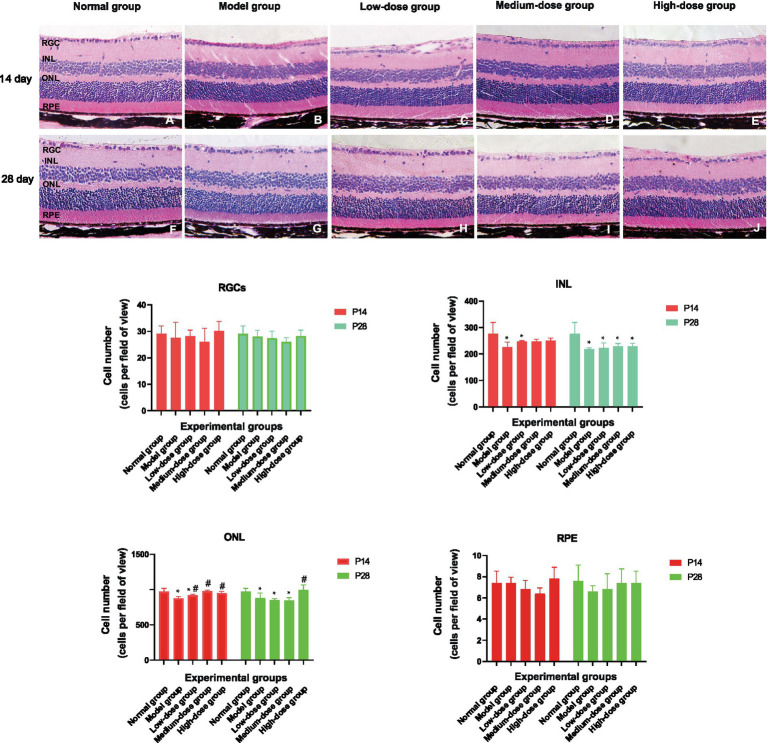
Hematoxylin and eosin (H&E) staining of retinal tissue (magnification, 400×). The figure shows representative histological images of retinal cross-sections from mice in each group at 14 and 28 days, illustrating the morphological changes and allowing for the measurement of retinal layer thicknesses. **(A–E)** Images from the normal control group, model group, low-dose group, medium-dose group, and high-dose group, respectively, at 14 days post-intervention. **(F–J)** Images from the normal control group, model group, low-dose group, medium-dose group, and high-dose group, respectively, at 28 days post-intervention. Statistical significance of measured layer thicknesses is indicated as follows: ^*^p < 0.05 vs. the normal control group; #*p* < 0.05 *vs.* the model group. RGC, retinal ganglion cell layer; INL, inner nuclear layer; ONL, outer nuclear layer; RPE, retinal pigment epithelium; H&E, hematoxylin and eosin.

H&E staining was used to evaluate the effect of Qiju Granule on retinal histopathological alterations. In the normal control group, the retina exhibited a normal and regular layered structure, with uniform cell morphology in each layer and a tightly integrated RPE layer (Figures 3A,F).

After 14 days of intervention, the model group showed obvious RPE atrophy and blurred structural integrity of Bruch’s membrane (Figure 3B). The low-dose (Figure 3C) and medium-dose (Figure 3D) Qiju Granule groups presented disorganized cells in the inner nuclear layer (INL) and outer nuclear layer (ONL), with focal thickening and thinning accompanied by loose arrangement in some regions. In contrast, the high-dose Qiju Granule group (Figure 3E) maintained an orderly retinal layered structure, and no vascular-like proliferative tissues were observed in Bruch’s membrane.

At 28 days post-treatment, the INL disorganization in the model group further deteriorated (Figure 3G). The retinal histopathological changes in each Qiju Granule-treated group were consistent with those observed at 14 days, with significant irregularities in the INL and ONL still evident.

Quantitative cell count analysis showed no significant differences in the number of retinal ganglion cells (RGCs) and RPE cells among all groups (*p* > 0.05). At the 14-day time point, compared with the normal control group, the INL cell count was significantly reduced in both the model group and the low-dose Qiju Granule group (*p* < 0.05). For the ONL, the model group, low-dose group, and medium-dose group all exhibited a significant decrease in cell count relative to the normal control group (*p* < 0.05). Conversely, the INL cell counts in the low-dose, medium-dose, and high-dose Qiju Granule groups were significantly higher than those in the model group (*p* < 0.05). At 28 days, the trend of INL cell counts remained consistent with that at 14 days. Notably, the ONL cell counts in the model group, low-dose group, and medium-dose group were still significantly lower than those in the normal control group (*p* < 0.05), while the high-dose Qiju Granule group showed a marked increase in ONL cell count compared with the model group (*p* < 0.05) (Figure 3).

### 3.4 The effect of Qiju Granule on cell apoptosis

**Figure 4:**
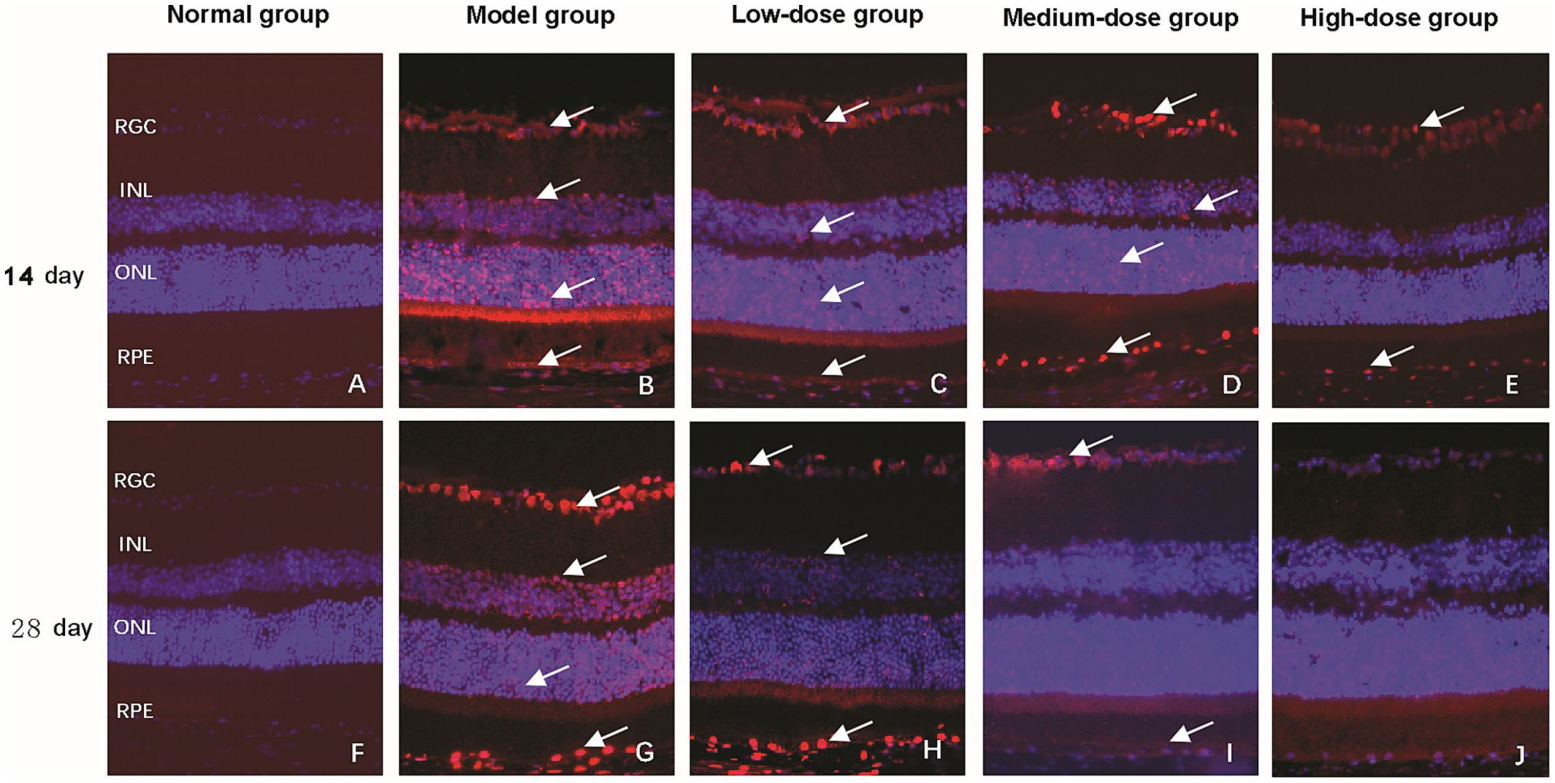
Detection of retinal cell apoptosis via TUNEL staining (magnification, 400×). The figure shows representative fluorescence microscopy images of retinal cross-sections from mice in each experimental group at 14 and 28 days. Apoptotic cells were visualized using terminal deoxynucleotidyl transferase dUTP nick end labeling (TUNEL) staining (red fluorescence), with nuclear counterstained with DAPI (blue fluorescence). **(A–E)** Images from the normal control group, model group, low-dose group, medium-dose group, and high-dose group, respectively, at 14 days post-intervention. **(F–J)** Images from the normal control group, model group, low-dose group, medium-dose group, and high-dose group, respectively, at 28 days post-intervention. White arrows indicate representative TUNEL-positive (apoptotic) cells. RGC, retinal ganglion cell layer; INL, inner nuclear layer; ONL, outer nuclear layer; RPE, retinal pigment epithelium; TUNEL, terminal deoxynucleotidyl transferase dUTP nick end labeling.

TUNEL staining was employed to evaluate the regulatory effect of Qiju Granule on retinal cell apoptosis. In the normal control group, only a minimal number of TUNEL-positive apoptotic cells were detected in the retina at both 14 and 28 days, indicating low basal apoptosis levels (Figures 4A,F).

At 14 days, the model group showed strong red fluorescent signals in the nuclei of RGCs, INL, ONL, and RPE layer, which is a typical hallmark of apoptotic cell death (Figure 4B). In contrast, all Qiju Granule-treated groups exhibited a distinct reduction in the number of apoptotic cells, with the distribution of positive signals consistent with that of the model group but with weaker fluorescence intensity (Figures 4C–E).

By 28 days, the model group (Figure 4G) and low-dose Qiju Granule group (Figure 4H) maintained a similar pattern of red fluorescence to that observed at 14 days, suggesting persistent high-level apoptosis. However, the medium-dose (Figure 4I) and high-dose (Figure 4J) Qiju Granule groups showed further reduced red fluorescent signals, which were mainly confined to the RGC layer, and no obvious TUNEL-positive apoptotic cells were detected in the RPE layer.

Statistical analysis revealed that after 14 days of intervention, the apoptosis rate in both the model group and all Qiju Granule-treated groups was significantly higher than that in the normal control group (*p* < 0.05). Notably, compared with the model group, all Qiju Granule-treated groups showed a marked reduction in apoptosis rate (*p* < 0.05). A clear dose-dependent effect was observed among the treatment groups: the high-dose and medium-dose groups exhibited superior efficacy in inhibiting apoptosis compared to the low-dose group, with the high-dose group achieving the most significant reduction in apoptosis rate (*p* < 0.05). This dose–response pattern was maintained consistently throughout the 28-day treatment period (Table 1).

**Table 1:** Comparison of retinal cell apoptosis rates (mean ± SD, *n* = 5).

Groups	Apoptosis rate	
	14 days	28 days
Normal control group	(0.44 ± 0.07)%	(0.44 ± 0.07)%
Model group	(56.16 ± 4.43)%^*^	(45.34 ± 1.92)%^*^
Low-dose group	(44.71 ± 0.88)%^*,#^	(11.80 ± 0.41)%^*,#^
Medium-dose group	(25.98 ± 1.11)%^*,#,&^	(7.81 ± 0.96)%^*,#^
High-dose group	(16.64 ± 1.62)%^*,#,&,Δ^	(6.74 ± 0.71)%^*,#,&^
*F*-value	101.217	298.942
*p*-value	0.000	0.000

Statistical significance denoted as: * vs. normal control group (*p* < 0.05); # vs. model group (*p* < 0.05); & vs. low-dose group (*p* < 0.05); Δ vs. medium-dose group (*p* < 0.05).

### 3.5 Effect of Qiju Granule on VEGF expression

**Figure 5:**
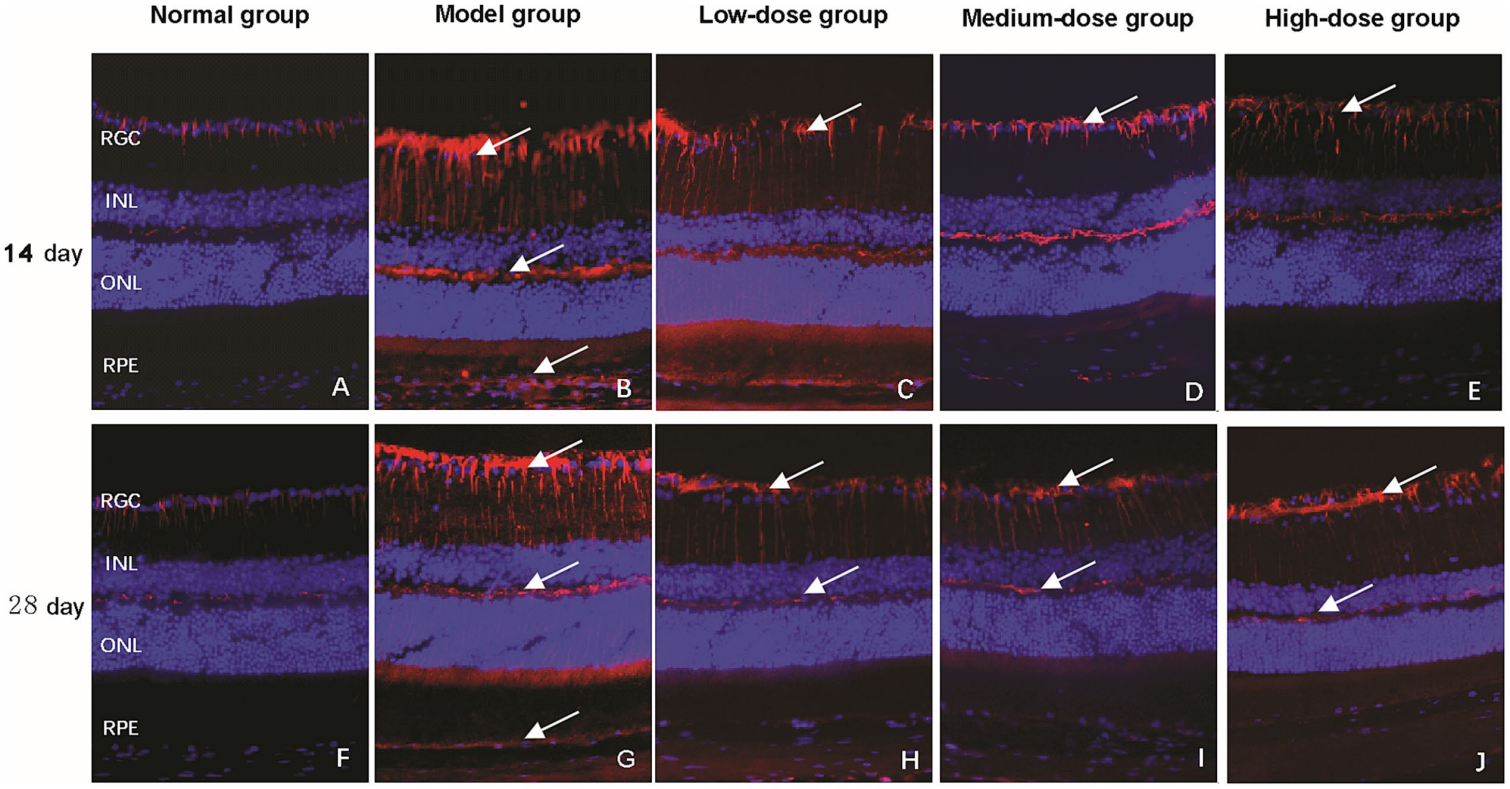
Effect of Qiju granule on vascular endothelial growth factor (VEGF) expression in the retina (magnification, 400×). The figure shows representative immunofluorescence images of retinal cross-sections from mice in each experimental group at 14 and 28 days. VEGF expression was visualized using a specific antibody (red fluorescence), and cell nuclei were counterstained with DAPI (blue fluorescence). **(A–E)** Images from the normal control group, model group, low-dose group, medium-dose group, and high-dose group, respectively, at 14 days post-intervention. **(F–J)** Images from the normal control group, model group, low-dose group, medium-dose group, and high-dose group, respectively, at 28 days post-intervention. White arrows indicate representative regions of positive VEGF expression. RGC, retinal ganglion cell layer; INL, inner nuclear layer; ONL, outer nuclear layer; RPE, retinal pigment epithelium; VEGF, vascular endothelial growth factor; DAPI, 4′,6-diamidino-2-phenylindole.

Immunofluorescence staining was used to detect the expression of VEGF, a key pro-angiogenic factor. In the normal control group, VEGF expression was minimal, with only weak fluorescent signals observed (Figure 5). After 14 days of intervention, the model group exhibited strong fluorescent signals, indicating a significant upregulation of VEGF expression. The low-dose Qiju Granule group showed a similar VEGF expression pattern to the model group, while the medium-dose group presented notably reduced fluorescent intensity. The high-dose group displayed even weaker VEGF fluorescence, with a more pronounced reduction, particularly in the RPE layer.

By 28 days post-treatment, the model group and low-dose Qiju Granule group maintained high VEGF expression levels consistent with the 14-day observations. In contrast, the medium-dose and high-dose Qiju Granule groups showed further attenuated fluorescent signals, which were mainly confined to the inner retina and RGC layer, and no detectable VEGF fluorescence was observed in the RPE layer (Figure 5). These results indicate that Qiju Granule can downregulate VEGF expression in a dose-dependent manner, with more significant inhibitory effects at medium and high doses.

### 3.6 Effect of Qiju Granule on CD31 expression

CD31, a specific marker of endothelial cells, was used to evaluate neovascularization. In the normal control group, CD31 expression was negligible, with almost no positive fluorescent signals detected. After 14 days, the model group showed a marked increase in red fluorescent signals, reflecting a significant upregulation of CD31 expression and enhanced endothelial cell activation. In contrast, the Qiju Granule-treated groups only exhibited weak CD31-positive signals, which were slightly detected in the RGC and RPE layers.

**Figure 6:**
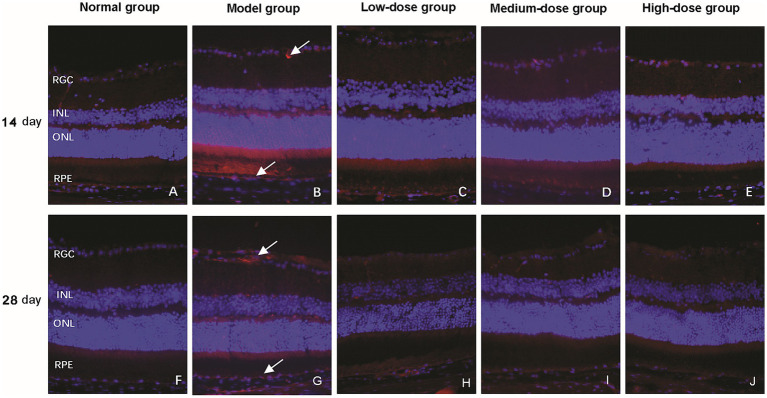
Effect of Qiju granule on CD31 expression in the retina (magnification, 400×). The figure shows representative immunofluorescence images of retinal cross-sections from mice in each experimental group at 14 and 28 days. CD31 expression was visualized using a specific antibody (red fluorescence), and cell nuclei were counterstained with DAPI (blue fluorescence). **(A–E)** Images from the normal control group, model group, low-dose group, medium-dose group, and high-dose group, respectively, at 14 days post-intervention. **(F–J)** Images from the normal control group, model group, low-dose group, medium-dose group, and high-dose group, respectively, at 28 days post-intervention. White arrows indicate representative regions of positive CD31 expression. RGC, retinal ganglion cell layer; INL, inner nuclear layer; ONL, outer nuclear layer; RPE, retinal pigment epithelium; CD31, platelet endothelial cell adhesion molecule-1, PECAM-1/CD31; DAPI, 4′,6-diamidino-2-phenylindole.

At 28 days, the model group showed widespread CD31 expression throughout the RGC layer, INL, ONL, and RPE layer, indicating progressive neovascularization. Conversely, the Qiju Granule-treated groups only presented a small number of weak CD31-positive signals, which were limited to the RPE layer, and the overall expression level was significantly lower than that in the model group (Figure 6). This suggests that Qiju Granule can inhibit CD31 expression and thereby suppress retinal neovascularization in AMD mice.

## 4 Discussion

This study investigated the therapeutic efficacy and mechanisms of Qiju Granule in a clinically relevant murine model of early-to-intermediate AMD induced by chronic light exposure and hydroquinone feeding. Our findings show that Qiju Granule preserves retinal structure and function by targeting two interrelated processes: retinal cell apoptosis and early angiogenic activation. These results provide new insights into the potential role of Qiju Granule in the management of AMD.

The doses of Qiju Granule used in this study were selected based on a combination of clinical translation and preclinical pharmacology principles. This conversion method is a well-established approach in preclinical TCM research, ensuring that the dose administered to mice is pharmacologically equivalent to the dose used in human clinical practice (30). The low dose (4.5 g/kg/day) and high dose (18 g/kg/day) were set at 0.5× and 2× the medium dose, respectively, a common dose-escalation design in TCM preclinical studies. This design allows for the evaluation of dose-dependent effects, which is critical for understanding the therapeutic window and potential efficacy of Qiju Granule in the context of AMD. Notably, the observed dose-dependent retinal protection in our study—with the high-dose group showing the most significant effects—aligns with the dose–response relationship predicted by this design. This consistency supports the validity of our dose selection strategy and reinforces the translational relevance of our findings.

Qiju Granule is a modern TCM formulation derived from two classical ophthalmic prescriptions. This dual heritage distinguishes it from single-origin formulations and likely underpins its multi-target pharmacological profile, which is well suited to AMD’s complex, multifactorial pathology. Traditionally, *Shihu Yeguang Pill* is used to “nourish liver and kidney yin, clear deficient fire, and improve vision,” and has historically been applied to retinal dystrophy and visual fatigue (15). By contrast, *Qiju Dihuang Pill* emphasizes “replenishing kidney essence and nourishing liver blood” and is traditionally used to alleviate age-related ocular dryness and age-related retinal decline (16, 31). This formulation distinction may explain its distinct efficacy profile relative to other TCM formulas studied in AMD, which often derive from a single classical prescription and may lack this synergistic design.

Comparison with our prior preclinical work reveals consistent retinal protective efficacy but a divergence in the mechanistic focus, rooted in the distinct pathological triggers of the two models and hypothesis-driven endpoint selection aligned with their respective disease contexts. In our earlier study (17), Qiju Granule mitigated retinal damage in a sodium iodate-induced rat model of dry AMD, where neurotrophic factor upregulation was identified as a key repair response to acute injury; in the present study, by contrast, our molecular analyses focused on VEGF and CD31, a choice guided by robust preclinical evidence linking hydroquinone—a critical smoking-related component of our model— to the dysregulation of pro-angiogenic signaling in early AMD (32, 33). Thus, the mechanistic differences likely reflect distinct pathological triggers rather than inconsistent efficacy and underscore the importance of clinically relevant models for dissecting Qiju Granule’s multi-target actions in progressive AMD.

Notably, although our findings support direct retinal actions of Qiju Granule—through effects on RPE cells and angiogenic signaling—we cannot exclude contributions from systemic metabolic effects, which warrant further study. TCM formulations are complex mixtures that can exert both local and systemic effects. Several Qiju Granule constituents (e.g., *Lycium barbarum* polysaccharides, chlorogenic acid, and puerarin) have been reported to modulate systemic pathways relevant to AMD pathogenesis. For instance, *Lycium barbarum* polysaccharides have been reported to increase systemic antioxidant capacity and suppress apoptosis (34, 35), which could indirectly reduce retinal oxidative stress by lowering systemic oxidative burden. Similarly, puerarin has been reported to regulate systemic glucose and lipid metabolism (36, 37), and chlorogenic acid to modulate peripheral inflammatory cytokines (38). Because systemic inflammation and metabolic dysregulation are risk factors for AMD progression (3, 39), these systemic effects may complement direct retinal actions. In addition, absorption and metabolism of Qiju Granule constituents may involve systemic that indirectly influence retinal physiology. For example, after oral administration, constituents may be metabolized by the liver and gut microbiota into bioactive metabolites that enter systemic circulation (40, 41). In addition, Qiju Granule’s traditional role in “nourishing liver and kidney” reflects TCM’s holistic framework linking systemic balance to ocular health; systemic regulation may indirectly support retinal homeostasis. Distinguishing direct retinal actions from systemic metabolic effects is important for translational development and can inform clinical positioning and formulation optimization.

Several limitations should be considered when interpreting these findings. First, although we observed reduced early angiogenic activation, the downstream signaling pathways involved (e.g., VEGFR2/STAT3) remain to be elucidated, including *in vitro* validation. Second, the study focused on early-to-intermediate stages of AMD; long-term studies are needed to determine whether Qiju Granule can prevent progression to late-stage neovascular AMD. Third, we did not measure systemic metabolic indicators or track the distribution of Qiju Granule’s bioactive components and their metabolites in retinal tissue relative to peripheral organs, which limits our ability to quantify the relative contributions of direct retinal action versus systemic metabolic effects to the observed therapeutic efficacy. This critical gap could be addressed in future research via targeted pharmacokinetic studies to quantify component and metabolite concentrations in both serum and retinal tissue, or by comparative efficacy studies of oral versus intravitreal Qiju Granule administration to isolate direct ocular effects from systemic modulation.

In conclusion, using a clinically relevant chronic model, this study advances understanding of Qiju Granule in AMD and suggests anti-angiogenic potential that has not been clearly attributed to its single classical precursors or related TCM formulations. These findings support integrating multiple classical prescriptions for complex ocular diseases and provide a mechanistic basis for developing Qiju Granule as a potential disease-modifying therapy for early-to-intermediate AMD. Future work to disentangle direct retinal versus systemic effects will refine mechanistic understanding and inform clinical development.

## 5 Conclusion

A multifactorial AMD mouse model was successfully established. Qiju Granule was found to exert a protective effect, mitigating damage to the RPE and photoreceptor cells, reducing cell death, and enhancing retinal function. Furthermore, Qiju Granule seemed to reduce the expression of factors related to neovascularization, suggesting its potential to prevent the progression to advanced AMD.

## Data Availability

The original contributions presented in the study are included in the article/supplementary material, further inquiries can be directed to the corresponding author.

## Glossary

AMD: Age-related macular degeneration
RPE: Retinal pigment epithelial
VEGF: Vascular endothelial growth factor
CD31: Platelet-endothelial cell adhesion molecule-1 PECAM-1/CD31
bFGF: Basic fibroblast growth factor
BDNF: Brain-derived neurotrophic factor
TCM: Traditional Chinese medicine
ERG: Electroretinogram
OCT: Optimal cutting temperature compound
HE: Hematoxylin–Eosin
INL: Inner nuclear layer
ONL: Outer nuclear layer
RGC: Retinal ganglion cell
TUNEL: Terminal deoxynucleotidyl transferase dUTP nick end labeling
PBS: Phosphate-buffered saline
BSA: Bovine serum albumin
ANOVA: Analysis of variance
LSD: Least significant difference
ROS: rReactive oxygen species
ARVO: Association for research in vision and ophthalmology
DAPI: 4′,6-diamidino-2-phenylindole
